# Liquid chromatography-tandem mass spectrometry analysis of pyrrolizidine alkaloids in Polish honey

**DOI:** 10.2478/jvetres-2026-0046

**Published:** 2026-08-27

**Authors:** Ewelina Kowalczyk

**Affiliations:** Department of Chemical Research of Food and Feed, National Veterinary Research Institute, 24-100 Puławy, Poland

**Keywords:** food contaminants, honey, LC-MS/MS, pyrrolizidine alkaloids, toxic plant secondary metabolites

## Abstract

**Introduction:**

Pyrrolizidine alkaloids (PAs) are among the most widespread naturally occurring toxins. Since they are primarily produced by flowering plants, there is a high likelihood of their transfer into honey. Given the toxic properties of PAs, their presence in honey can significantly compromise the safety of the product. Therefore, monitoring the occurrence of PAs in honey is essential.

**Material and Methods:**

A total of 65 honey samples collected in Poland were analysed for the presence of 42 PAs. The samples included multifloral, acacia, rapeseed and forest honeys. Pyrrolizidine alkaloids were quantified using liquid chromatography coupled with tandem mass spectrometry (LC-MS/MS).

**Results:**

Quantifiable amounts of PAs were detected in 10 of the honey samples. At least one of the monitored PAs was present in 39 samples, although most were below the method’s limit of quantification. The highest concentration observed was 46.9 μg/kg.

**Conclusion:**

A newly validated LC-MS/MS method was successfully applied to determine PAs in honey from Poland. Most of the analysed samples were free of the monitored PAs, confirming the general safety of Polish honey.

## Introduction

Honey, one of the oldest natural food products, has long been valued for its nutritional and medicinal properties. Its complex chemical composition contains a wide range of bioactive compounds, including vitamins, minerals, and enzymes. In recent years, increasing consumer awareness of food safety and health risks has intensified research into undesirable substances in honey (1). Among them, pyrrolizidine alkaloids (PAs) have attracted particular concern.

Pyrrolizidine alkaloids are natural plant-derived toxins produced by approximately 6,000 species, or around 3% of all flowering plants (9). They are considered the most widespread group of naturally occurring plant toxins, and pose potential risks to both human and animal health. Plants which produce PAs occur across diverse climates, with major sources belonging to the families Boraginaceae, Asteraceae, Fabaceae and Apocynaceae (11, 21, 23). In Poland, common representatives include groundsel (*Senecio vulgaris*), ragwort (*Senecio jacobaea*), comfrey (*Symphytum officinale*), viper’s bugloss (*Echium vulgare*) and borage (*Borago officinalis*). Often regarded as invasive weeds, these plants are abundant in meadows, pastures, wastelands and cultivated fields, thereby posing a risk of food and feed contamination (2).

The biosynthesis of PAs varies by species, but they are typically produced in roots and transported throughout the plant. Inflorescences generally contain the highest concentrations, followed by leaves and then stems (20). Alkaloid levels may range from trace amounts to several percent of the plant’s dry mass, with composition and concentration influenced by environmental conditions, climate and developmental stage (9). To date, around 700 PA structures have been identified, and the number continues to grow (8). They occur as tertiary bases (free bases) and N-oxides, both of which exhibit toxic properties (9, 8, 18).

Pyrrolizidine alkaloids act as pro-toxins, becoming toxic only after metabolic activation in the liver. Their transformation into reactive metabolites leads to DNA adduct formation and subsequent cell and tissue damage, primarily affecting the liver and lungs. This process is associated with mutagenesis, carcinogenesis, and, in severe cases, liver failure (4, 12, 24). Although toxic potency varies among compounds, the European Food Safety Authority (EFSA) has concluded that all 1,2-unsaturated PAs follow the same metabolic pathway, producing genotoxic and carcinogenic pyrroles (9). Even single low-dose exposures can be harmful, and the International Agency for Research on Cancer (IARC) has classified several PAs as “possibly carcinogenic to humans” (Group 2B) (9, 10). Documented cases of poisoning by PA-contaminated food, some fatal, highlight the risks to human health (11).

Honey is particularly vulnerable to PA contamination. Bees foraging on PA-producing plants collect nectar and pollen containing these toxins, which are subsequently introduced into honey (15). Because bees are not selective in nectar sources, PA contamination often goes undetected by standard quality control testing (11). Apiaries located near PA-rich vegetation are therefore at higher risk of producing contaminated honey.

Given the health risks, risk management strategies have been introduced by international authorities. In 2020, the European Union established maximum permissible PA levels in certain products, including pollen, pollen-based foods and supplements, for which 500 μg/kg was set (7). Although specific regulations concerning PAs in honey are still lacking, increasing awareness of the risks has led to greater attention being paid to monitoring of these substances in a broader range of bee products. Levels of PA contamination in honey depend on multiple factors, such as hive location, production season, the flowering period of PA-containing plants and weather conditions. Regular monitoring of honey is therefore essential to ensure consumer safety.

The aim of this study was to assess the occurrence of 42 PAs in Polish honey using liquid chromatography-tandem mass spectrometry (LC-MS/MS), with the goal of evaluating potential risks to consumer health and supporting future regulatory efforts.

## Material and Methods

### Chemicals and reagents

Ethyl acetate of gaschromatography grade was purchased from Merck (Darmstadt, Germany). Sulphuric acid (95%) and ammonia in 25% solution was from POCH (now Avantor Performance Materials, Gliwice, Poland). Ammonium carbonate was obtained from Sigma-Aldrich (St. Louis, MO, USA). Acetonitrile and methanol were ordered from J.T. Baker (now Avantor Performance Materials, Deventer, the Netherlands). Water came from Milli-Q water purification system (Millipore Sigma, Burlington, MA, USA). Mixed mode Oasis MCX cation exchange cartridges for solid-phase extraction (SPE) of 500 mg bed weight and 6 mL volume were provided by Waters (Milford, MA, USA). Pyrrolizidine alkaloid standards were purchased from LGC Standards (Teddington, UK) as a mix of europine, europine N-oxide, intermedine, intermedine N-oxide, lycopsamine, lycopsamine N-oxide, heliotrine, heliotrine N-oxide, retrorsine, retrorsine N-oxide, seneciphylline, seneciphylline N-oxide, senecivernine, senecivernine N-oxide, senecionine, senecionine N-oxide, echimidine, echimidine N-oxide, senkirkine, lasiocarpine and lasiocarpine N-oxide. Additionally, individual standards of monocrotaline, erucifoline, erucifoline N-oxide, jacobine, jacobine N-oxide, jaconine, jaconine N-oxide, trichodesmine, rinderine, rinderine N-oxide, echinatine, echinatine N-oxide, indicine, indicine N-oxide, integerrimine, integerrimine N-oxide, heliosupine, heliosupine N-oxide, usaramine, usaramine N-oxide and spartioidine were purchased from Phytolab (Vestenbergsgreuth, Germany).

### Standard solutions

Standard stock solutions of the individual compounds were prepared in methanol at a concentration of 1,000 μg/mL. The 21-PA mix was purchased at the concentration of 100 μg/mL and used unchanged. Mixed working standard solutions at a concentration of 10 μg/mL were prepared by adding an appropriate amount of each single stock solution and the 21-PA mix. The mixed solution was further diluted with methanol to obtain concentrations of 1 μg/mL and 0.1 μg/mL. All solutions were stored <–18°C and were stable for 12 months.

### Honey samples

These were collected as regular veterinary inspection procedures or sent directly by apiarists for privately commissioned testing. In total 65 honey samples were analysed. The analysed honeys were of Polish origin, and at least one sample was taken from each of the 16 voivodeships: 5 multifloral or rapeseed samples were from Dolnośląskie; 1 acacia sample was from Kujawsko-Pomorskie; 15 multifloral, rapeseed or acacia samples were from Lubelskie; 4 multifloral or rapeseed samples were from Lubuskie; 3 multifloral samples were from Łódzkie; 4 multifloral, honeydew or forest honey samples were from Małopolskie; 5 multifloral, rapeseed, acacia or linden samples were from Mazowieckie; 9 forest, multifloral or rapeseed samples were from Opolskie; 1 multifloral sample was from Podkarpackie; 4 multifloral or rapeseed samples were from Podlaskie; 2 rapeseed samples were from Pomorskie; 2 multifloral samples were from Śląskie; 3 multifloral or rapeseed samples were from Świętokrzyskie; 1 sample of unknown type was from Warmińsko-Mazurskie; 3 rapeseed samples were from Wielkopolskie; and 3 rapeseed samples were from Zachodniopomorskie.

### Sample preparation

If necessary, honey samples were heated in a water bath at 40–45°C and homogenised. From each sample, 10 g of honey was weighed into a 50 mL polypropylene tube and diluted with 20 mL of 0.05 M sulfuric acid. All samples were shaken on a horizontal shaker until completely dissolved. Subsequently, the samples were subjected to centrifugation for 10 min at 4,000 × *g*. The resulting extracts were subjected to SPE using Oasis MCX mixed-mode cation exchange cartridges (Waters). The cartridges were preconditioned with 6 mL of methanol followed by 6 mL of 0.05 M H_2_SO_4_. After conditioning, 10 mL of the extract was loaded onto the cartridges. The washing step included sequential rinsing with 9 mL of purified water and 9 mL of methanol, followed by vacuum drying for 2 min. Alkaloids were eluted using 10 mL of a solvent mixture composed of ethyl acetate, methanol, acetonitrile, ammonia solution and triethylamine in a ratio of 8:1:1:0.5:0.1 (v/v). After evaporation under a stream of nitrogen, the residues were reconstituted in 0.4 mL of purified water and 0.4 mL of methanol. The solutions were then filtered through a 0.2 μm polyvinylidene fluoride filter and analysed.

### Instrumental analysis

Chromatographic separation was achieved on an ExionLC Series UHPLC system (Sciex, Framingham, MA, USA) equipped with a Gemini NX-C18 column, 3 μm, 100 × 3 mm (Phenomenex, Torrance, CA, USA). The mobile phase consisted of 5 mM ammonium carbonate in water (solvent A) and a mixture of acetonitrile and methanol (2:1, v/v) (solvent B). Detection of all of the analytes was accomplished using a Triple Quad 5500+ triple-quadrupole mass spectrometer (Sciex) operated in multiple reaction monitoring mode. A Turbo V (Sciex) source was used with an electrospray ionization probe in positive polarity, and the parameters were optimised for optimal sensitivity. The ion source temperature and the ion spray voltage were set to 550°C and 4,500 V, respectively. Ion source gases 1 and 2 were both set to 50 psi. Nitrogen was used as the collision gas and the curtain gas at pressures of 9 psi and 30 psi, respectively. All other detection settings (precursor ions (m/z), product ions (m/z), declustering potential, entrance potential, collision energy and collision cell exit potential) are presented in Table 1. Analyst software (Sciex) was used for data acquisition, and the data was processed with Sciex OS software.

**Table 1.** Optimised parameters of ion source

Compound	Q1 mass (Da)	Q3 mass (Da)	DP (V)	EP (V)	CE (V)	CXP (V)
Echimidine	398.2	120.1*/220.1/238.1	121	10	33/25/43	14/12/12
Echimidine N-oxide	414.2	254.2*/352.2/220.2	126	10	43/35/55	14/18/11
Echinatine	300.7	138.2*/156.2/120.2	104	10	31/37/39	18/8/18
Echinatine N-oxide	316.3	172.2*/111.2/94.2	110	10	39/51/53	8/14/16
Erucifoline	350.2	120.3*/138.1/220.1	152	10	38/38/45	14/16/12
Erucifoline N-oxide	366.2	118.0*/94.0/120.2	150	10	58/40/40	11/15/15
Europine	330.2	138.2*/254.1/156.3	127	10	30/27/40	16/13/10
Europine N-oxide	346.2	172.1*/256.3/111.2	60	10	42/35/57	21/12/12
Heliosupine	398.0	120.2*/220.2/336.2	101	10	37/29/27	16/12/16
Heliosupine N-oxide	414.0	254.3*/94.2/120.2	122	10	41/61/45	14/12/18
Heliotrine	314.1	138.2*/156.2/94.2	114	10	29/37/45	18/20/16
Heliotrine N-oxide	330.2	172.0*/138.1/94.1	158	10	37/38/54	21/16/12
Indicine	300.4	94.2*/156.3/138.0	130	10	35/38/28	11/15/16
Indicine N-oxide	316.4	172.3*/138.2/94.2	120	10	37/37/53	8/18/18
Intermedine	300.3	94.3*/156.2/138.3	135	10	35/38/27	11/17/12
Intermedine N-oxide	316.2	172.2*/94.0/111.3	130	10	38/51/53	15/11/13
Integerrimine	336.2	120.1*/94.0/308.0	150	10	38/42/36	15/11/16
Integerrimine N-oxide	352.2	118.1*/94.1/136.2	149	10	60/39/42	11/14/12
Jacobine	352.2	120.1*/280.1/155.2	136	10	33/39/39	14/14/8
Jacobine N-oxide	368.2	296.2*/120.3/94.0	137	10	46/34/56	15/15/11
Jaconine	388.1	352.2*/94.1/120.0	66	10	41/49/49	18/12/18
Jaconine N-oxide	404.0	118.1*/94.2	100	10	43/69	16/18
Lasiocarpine	412.2	120.2*/220.3/336.2	81	10	35/27/27	16/12/18
Lasiocarpine N-oxide	428.1	254.2*/120.2/94.2	130	10	41/45/57	12/18/12
Lycopsamine	300.3	94.2*/156.1/138.1	135	10	36/38/29	11/16/16
Lycopsamine N-oxide	316.2	172.3*/94.2/111.2	142	10	56/38/51	11/15/13
Monocrotaline	326.2	120.2*/121.1/237.1	151	10	45/39/33	14/14/12
Retrorsine	352.2	120.2*/138.4/324.0	150	10	39/39/38	7/6/16
Retrorsine N-oxide	368.2	94.1*/120.0/136.0	124	10	62/42/43	11/15/18
Rinderine	300.4	138.2*/156.3/94.2	156	10	31/37/45	18/8/16
Rinderine N-oxide	316.4	172.3*/111.1/94.2	110	10	39/51/53	8/20/14
Seneciphylline	334.2	120.1*/306.3/94.1	146	10	37/35/41	14/16/14
Seneciphylline N-oxide	350.2	94.1*/118.1/136.3	140	10	57/40/42	11/14/15
Senecionine	336.2	120.1*/308.2/138.1	151	10	39/37/39	12/16/16
Senecionine N-oxide	352.1	118.0*/94.3/136.1	131	10	40/61/45	14/11/16
Senceivernine	336.2	308.2*/120.1/138.2	161	10	37/39/41	16/14/6
Senecivernine N-oxide	352.1	94.1*/120.2/136.0	155	10	58/45/43	11/14/14
Senkirkine	366.2	168.2*/150.2/122.2	126	10	39/37/43	8/8/16
Spartioidine	334.2	120.1*/94.2/138.3	142	10	36/43/39	14/11/12
Trichodesmine	354.3	222.4*/120.1/138.1	150	10	38/46/46	11/6/18
Usaramine	352.2	120.0*/94.0/138.0	151	10	38/44/39	19/16/16
Usaramine N-oxide	368.2	94.0*/120.0/136.2	150	10	63/42/43	16/19/17

Q1 – precursor ion; Q3 – product ion; DP – declustering potential; EP – entrance potential; CE – collision energy; CXP – collision cell exit potential;

* – quantifier ion

### Method validation

The method was subjected to validation according to the “Analytical quality control and method validation procedures for pesticide residues analysis in food and feed” in SANTE 11312/2021 (5). During the validation process, the parameters assessed included linearity, recovery, repeatability, reproducibility, matrix effect, selectivity, limit of detection (LOD), limit of quantification (LOQ) and uncertainty.

Honey samples with no detectable levels of the monitored PAs were utilised as blank samples for preparing fortified samples used in validation. The method's linearity was evaluated through the analysis of matrix calibration curves and the determination of the R^2^ coefficient. Blank honey samples were spiked at concentrations of 1, 2.5, 5, 10, 20, 50 and 100 μg/kg prior to the extraction process. Linearity was confirmed if the coefficient of determination (R^2^) exceeded 0.98. An additional criterion for assessing linearity was the precision of back-calculated concentrations, which had to fall within a 20% margin of error. To evaluate the selectivity of the method, a series of blank honey samples was analysed to identify any potential interferences from endogenous matrix components at the retention times of the monitored alkaloids. For assessment of the matrix effect, blank honey samples were spiked with standard solutions after the SPE cleanup, while corresponding standard solutions were also prepared in pure solvents at the same concentrations. The matrix effect (%) was calculated as the ratio of the analyte peak area in the matrix-matched standard to the peak area in the solvent-based standard, multiplied by 100. The limit of detection was determined based on a signal-to-noise ratio of 3. The lowest validation level for which parameters such as precision and accuracy were determined was chosen as the limit of quantification. Recovery, repeatability and within-laboratory reproducibility were assessed by analysing three replicate blank samples spiked at each of three concentration levels: 1, 10 and 100 μg kg^−1^. Recovery (%) was calculated using the formula:

%Recovery=(measured concentration / fortification level) × 100.

Repeatability was assessed by calculating the coefficient of variation (CV%) at each tested concentration level. Within-laboratory reproducibility was evaluated by analysing two additional sets of spiked blank honey samples – prepared at the same concentration levels used for repeatability – on different days using the same analytical instrument. The CV% values obtained from these measurements were used to assess reproducibility. Measurement uncertainty was estimated by identifying and quantifying all relevant sources of uncertainty throughout the entire analytical procedure, in accordance with guidelines from Eurachem and the Co-Operation on International Traceability in Analytical Chemistry (CITAC) (6). The expanded uncertainty was reported as a percentage, using a coverage probability of 95% (P-value = 0.05) and a coverage factor of k = 2.

## Results

The sample preparation procedure was adapted from the previous studies on PAs in honey, with a slight modification of the SPE elution mixture to enhance the recoveries of all N-oxide forms (16, 17). In the present study, the concentration of ammonia was increased fivefold compared to the previously applied method. Good chromatographic separation of most of the isomers was achieved thanks to application of 5 mM ammonia carbonate and mixture of methanol and acetonitrile (2:1, v/v) as the mobile phases.

The presented method was validated in house. Among the assessed parameters were linearity in the concentration range 1–100 μg/kg, matrix effect, selectivity, recovery, repeatability, reproducibility, LOD, LOQ and uncertainty. All validation results are presented in Tables 2 and 3.

**Table 2.** Validation results of recovery, repeatability and reproducibility

Compound	Recovery (1 μg kg^−1^ spike) (%)	Recovery (10 μg kg^−1^ spike) (%)	Recovery (100 μg kg^−1^ spike) (%)	Repeat ability (1 μg kg^−1^ spike) (CV, %)	Repeat ability (10 μg kg^−1^ spike) (CV, %)	Repeat ability (100 μg kg^−1^ spike) (CV, %)	Reproducibility (1 μg kg^−1^ spike) (CV, %)	Reproducibility (10 μg kg^−1^ spike) (CV, %)	Reproducibility (100 μg kg^−1^ spike) (CV, %)
Echimidine	106.7	110.0	99.0	3.6	2.0	5.2	14.4	2.9	4.9
Echimidine N-oxide	105.2	107.9	97.1	5.4	3.1	1.2	19.0	8.6	7.5
Erucifoline	103.1	117.6	104.1	2.0	1.4	2.0	19.2	5.4	7.2
Erucifoline N-oxide	111.2	109.7	97.8	3.8	5.6	2.7	13.7	16.3	9.1
Europine	109.8	108.6	93.9	3.2	2.3	5.7	10.7	3.9	6.0
Europine N-oxide	103.1	110.2	91.3	5.5	6.0	2.9	18.0	11.8	10.5
Heliosupine	118.1	112.3	97.2	2.8	1.8	2.3	14.9	2.9	5.1
Heliosupine N-oxide	105.5	108.2	95.9	3.1	3.2	4.8	13.9	6.4	5.2
Heliotrine	105.4	111.4	98.9	4.2	2.4	5.6	17.8	4.2	5.4
Heliotrine N-oxide	106.6	107.9	94.8	4.1	1.8	5.5	16.2	7.4	6.7
Intermedine	106.5	110.6	98.8	3.6	2.7	4.5	14.2	4.9	4.0
Intermedine N-oxide	110.3	111.1	93.1	4.1	2.1	4.8	10.5	7.3	7.0
Integerrimine	101.0	116.0	103.8	4.7	3.9	3.3	19.9	4.3	6.3
Integerrimine N-oxide	107.8	107.3	95.4	3.9	4.0	2.9	13.5	9.7	4.7
Jacobine	102.9	118.2	103.6	5.1	2.7	2.9	19.6	6.6	8.9
Jacobine N-oxide	106.1	107.4	92.9	8.7	5.4	6.1	13.3	8.9	6.4
Jaconine	95.8	118.3	109.4	5.6	4.4	4.3	19.3	7.0	8.7
Jaconine N-oxide	101.6	109.7	99.5	4.0	11.6	3.5	19.8	11.2	6.3
Lasiocarpine	105.7	112.8	90.4	7.3	4.4	2.5	18.4	6.8	5.8
Lasiocarpine N-oxide	107.2	111.2	94.2	2.8	1.9	4.1	16.8	6.6	7.2
Lycopsamine	103.3	116.6	99.8	5.0	4.5	3.0	19.8	4.1	4.4
Lycopsamine N-oxide	107.3	109.5	92.2	8.7	3.9	3.4	13.6	13.5	7.6
Monocrotaline	98.3	116.4	105.5	8.3	2.6	3.2	18.5	3.2	6.6
Retrorsine	103.6	110.1	96.4	4.2	3.7	3.3	14.9	3.2	4.2
Retrorsine N-oxide	105.6	106.6	92.8	5.9	3.8	2.5	16.8	9.8	7.7
Rinderine	105.5	114.7	101.8	2.4	2.8	2.0	17.1	4.3	4.2
Rinderine N-oxide	107.9	110.6	93.6	6.2	3.3	5.1	14.9	6.3	6.2
Seneciphylline	104.6	114.3	97.8	4.1	3.2	3.3	19.1	5.3	6.0
Seneciphylline N-oxide	105.1	107.9	96.1	1.6	2.8	2.5	18.7	11.2	7.7
Senecionine	102.8	112.9	100.8	3.6	1.7	1.9	19.4	4.0	6.5
Senecionine N-oxide	111.1	106.4	93.0	2.9	1.7	0.8	12.5	10.5	6.9
Senceivernine	103.8	114.6	99.6	6.5	2.6	2.5	13.4	4.5	6.4
Senecivernine N-oxide	106.5	106.9	95.4	5.6	6.2	4.1	15.1	10.6	8.1
Senkirkine	100.6	110.9	100.5	7.4	4.6	3.7	13.4	6.2	6.3
Spartioidine	106.1	117.1	100.4	5.0	4.1	2.6	20.0	4.9	6.8
Trichodesmine	100.2	115.0	107.6	10.3	1.6	2.5	17.1	3.5	6.4
Usaramine	105.6	114.8	98.9	4.4	4.3	3.0	17.8	5.4	6.3
Usaramine N-oxide	108.1	106.0	94.6	11.1	3.0	3.5	16.0	8.6	6.8

**Table 3.** Validation results of linearity, matrix effect, uncertainty and limit of detection (LOD)

Compound	Linearity (R^2^)	Matrix effect (%)	Uncertainty (%)	LOD (μg kg^−1^)
Echimidine	0.993	106.4	32.5	0.01
Echimidine N-oxide	0.995	97.0	40.5	0.01
Erucifoline	0.990	109.6	40.0	0.03
Erucifoline N-oxide	0.994	113.8	38.7	0.04
Europine	0.995	106.9	29.5	0.01
Europine N-oxide	0.990	127.3	37.5	0.02
Heliosupine	0.997	97.8	33.9	0.01
Heliosupine N-oxide	0.996	105.7	30.7	0.03
Heliotrine	0.995	110.7	38.3	0.01
Heliotrine N-oxide	0.994	99.5	35.9	0.01
Intermedine	0.994	107.5	32.0	0.01
Intermedine N-oxide	0.992	94.7	29.8	0.02
Integerrimine	0.996	102.8	40.1	0.02
Integerrimine N-oxide	0.997	96.9	31.7	0.06
Jacobine	0.997	104.8	40.7	0.08
Jacobine N-oxide	0.997	93.9	29.9	0.01
Jaconine	0.996	104.9	38.4	0.02
Jaconine N-oxide	0.994	110.1	40.9	0.02
Lasiocarpine	0.996	105.0	39.5	0.01
Lasiocarpine N-oxide	0.992	102.3	37.4	0.01
Lycopsamine	0.994	109.2	39.9	0.01
Lycopsamine N-oxide	0.990	119.8	31.6	0.01
Monocrotaline	0.996	108.2	38.3	0.01
Retrorsine	0.995	99.0	31.5	0.03
Retrorsine N-oxide	0.990	96.6	36.4	0.03
Rinderine	0.996	101.4	36.9	0.01
Rinderine N-oxide	0.993	100.9	34.5	0.02
Seneciphylline	0.995	102.0	38.5	0.01
Seneciphylline N-oxide	0.994	99.7	39.8	0.01
Senecionine	0.995	104.0	39.2	0.03
Senecionine N-oxide	0.995	97.8	34.0	0.04
Senceivernine	0.994	100.0	30.7	0.03
Senecivernine N-oxide	0.994	97.1	33.7	0.03
Senkirkine	0.997	98.2	27.5	0.01
Spartioidine	0.994	101.3	40.2	0.02
Trichodesmine	0.998	114.9	35.1	0.01
Usaramine	0.996	99.3	38.4	0.06
Usaramine N-oxide	0.995	104.9	36.6	0.07

The recoveries ranged from 90.4% to 118.3%, depending on the compound and validation level. The CV for repeatability ranged from 0.8% to 11.6%, and the CV for reproducibility from 2.9% to 20.0%. Regarding linearity, the calibration curves for all compounds had a coefficient of determination (R^2^) greater than 0.98. Additionally, back-calculated concentrations did not deviate by more than ±20%. The matrix effect was mainly observed as ion enhancement, ranging from 93.9% to 127.3%, with the highest value recorded for europine N-oxide. The limit of detection, defined as a signal-to-noise ratio of 3, ranged from 0.01 to 0.08 μg/kg, depending on the compound. The lowest validation level, 1 μg/kg, was adopted as the LOQ for all analysed compounds. Table 3 presents the highest uncertainty values obtained for each compound, regardless of the validation level.

Out of the 65 honey samples tested, 39 contained at least one of the analysed compounds. However, in most cases, the concentrations were very low, falling below the method’s LOQ (<1 μg/kg). In 23 samples, more than one of the monitored compounds was detected. The most commonly found alkaloids were spartioidine and seneciphylline, both belonging to the group of macrocyclic diesters. Other frequently detected alkaloids included open-chain monoester compounds from the intermedine group: rinderine/echinatine, intermedine and lycopsamine/indicine, as well as their N-oxide forms. The presence of macrocyclic diester senecionine and senecivernine, along with open-chain diester echimidine and heliosupine and their oxides was also observed.

Only 10 samples (15.4%) contained alkaloids at concentrations above the method’s LOQ. The measured concentrations, expressed as the sum of the detected alkaloids, ranged from 1.0 to 46.9 μg/kg. The highest concentration among all the compounds was of seneciphylline at 21.3 μg/kg, followed by those of heliosupine N-oxide at 18.9 μg/kg, heliosupine at 13.2 μg/kg, rinderine/echinatine at 12.5 μg/kg and echimidine at 9.1 μg/kg (Tables 4 and 5). Most of the quantified levels, however, were low, very often in the range from 1.0 to 2.0 μg/kg. The mean content of PAs was calculated as 12.7 μg/kg, and the median was evaluated as 7.8 μg/kg.

**Table 4.** Concentrations of open-chain pyrrolizidine alkaloids (PAs) in μg/kg in positive samples of honey

Voiv.	Echimidine	Heliosupine	Echimidine N-oxide	Heliosupine N-oxide	Intermedine	Lycopsamine/indicine	Rinderine / echinatine	Intermedine N-oxide	Lycopsamine N-oxide/indicine N-oxide	Rinderine N-oxide / echinatine N-oxide
LU										
LU										
LU		< LOQ				< LOQ				< LOQ
LU	9.1				< LOQ					
LU										
LU										
LU										
LU										
LU					1.3	1.2	12.5			2.8
LU						< LOQ				< LOQ
LU									< LOQ	< LOQ
LU										
LU							< LOQ			
DS										
MP										
MP							< LOQ			
MP							< LOQ			
MP						< LOQ	< LOQ			
OP										
OP						< LOQ				
OP										
OP										< LOQ
ZP										
ZP		< LOQ								
ZP						< LOQ		< LOQ	< LOQ	< LOQ
WM						< LOQ				< LOQ
WP					< LOQ	< LOQ	< LOQ			
WP										
ŁD							< LOQ			
SL										< LOQ
SL									< LOQ	
LS						< LOQ				
LS										
PL						< LOQ				
MZ					< LOQ	< LOQ				
MZ	2.1		< LOQ		< LOQ	< LOQ	< LOQ	< LOQ	< LOQ	
MZ	4.2	13.2			< LOQ	< LOQ	< LOQ			
MZ	3.6	7.5	7.1	18.9	< LOQ	1.3	< LOQ	6.0	2.5	
PL	< LOQ									

Voiv.– voivodeship; LU – Lubelskie; DS – Dolnośląskie; MP – Małopolskie; OP – Opolskie; ZP – Zachodnio-Pomorskie; WM – Warmińsko-Mazurskie; WP – Wielkopolskie; ŁD – Łódzkie; SL – Śląskie; LS – Lubuskie; PL – Podlaskie; MZ – Mazowieckie; PK – Podkarpackie; LOQ – limit of quantification

**Table 5.** Concentrations of macrocyclic pyrrolizidine alkaloids (PAs) in μg/kg in positive samples of honey and the total PA content in μg/kg representing the sum of open-chain and macrocyclic PAs

Voivodeship	Seneciphylline	Spartioidine	Seneciphylline N-oxide	Senecionine	Senecivernine	Total PA content
LU		< LOQ				0.0
LU		< LOQ				0.0
LU					1.0	1.0
LU	1.7			1.0	1.0	12.8
LU		< LOQ				0.0
LU		< LOQ				0.0
LU		< LOQ				0.0
LU	< LOQ					0.0
LU	< LOQ					17.8
LU	< LOQ	< LOQ				0.0
LU		< LOQ				0.0
LU		< LOQ				0.0
LU		< LOQ				0.0
DS		< LOQ				0.0
MP		< LOQ				0.0
MP	< LOQ					0.0
MP	21.3		2.3			23.6
MP	< LOQ	< LOQ				0.0
OP	< LOQ					0.0
OP	< LOQ	< LOQ				0.0
OP		< LOQ				0.0
OP		< LOQ				0.0
ZP		< LOQ				0.0
ZP		< LOQ				0.0
ZP		< LOQ				0.0
WM	< LOQ	1.1				1.1
WP		< LOQ				0.0
WP	< LOQ	< LOQ				0.0
ŁD		< LOQ				0.0
SL		< LOQ				0.0
SL						0.0
LS	< LOQ	1.8			1.1	2.9
LS		< LOQ				0.0
PL					< LOQ	0.0
MZ		1.5				1.5
MZ						2.1
MZ	< LOQ	< LOQ	< LOQ	< LOQ		17.4
MZ						46.9
PL						0.0

LU – Lubelskie; DS – Dolnośląskie; MP – Małopolskie; OP – Opolskie; ZP – Zachodnio-Pomorskie; WM – Warmińsko-Mazurskie; WP – Wielkopolskie; ŁD – Łódzkie; SL – Śląskie; LS – Lubuskie; PL – Podlaskie; MZ – Mazowieckie; PK – Podkarpackie; LOQ – limit of quantification

## Discussion

Pyrrolizidine alkaloids are toxins that have received increasing attention in recent years. Numerous analytical methods have been published in the literature; however, none of them have so far achieved complete separation of all the compounds listed in Regulation (EU) 2020/2040 (7). The most challenging group of isomers includes intermedine, lycopsamine, indicine, rinderine, echinatine and their corresponding N-oxides. In the present study, it was possible to achieve separation of intermedine, lycopsamine and rinderine. However, indicine coeluted with lycopsamine, and echinatine could not be separated from rinderine. The same separation pattern was observed for the corresponding N-oxides. For this reason, indicine, echinatine and their N-oxides were excluded from the standard mix and acquisition method, and their potential presence was quantified as lycopsamine and rinderine, respectively. A similar approach was used for the corresponding N-oxides. Other challenging isomers, including senecionine, senecivernine and integerrimine, were successfully separated in both their free-base forms and as the corresponding N-oxides (Fig. 1). All remaining isomers were either fully resolved or only partially co-eluting; in the latter case, sufficient chromatographic resolution was achieved to allow reliable quantification as distinct peaks.

**Fig. 1.**
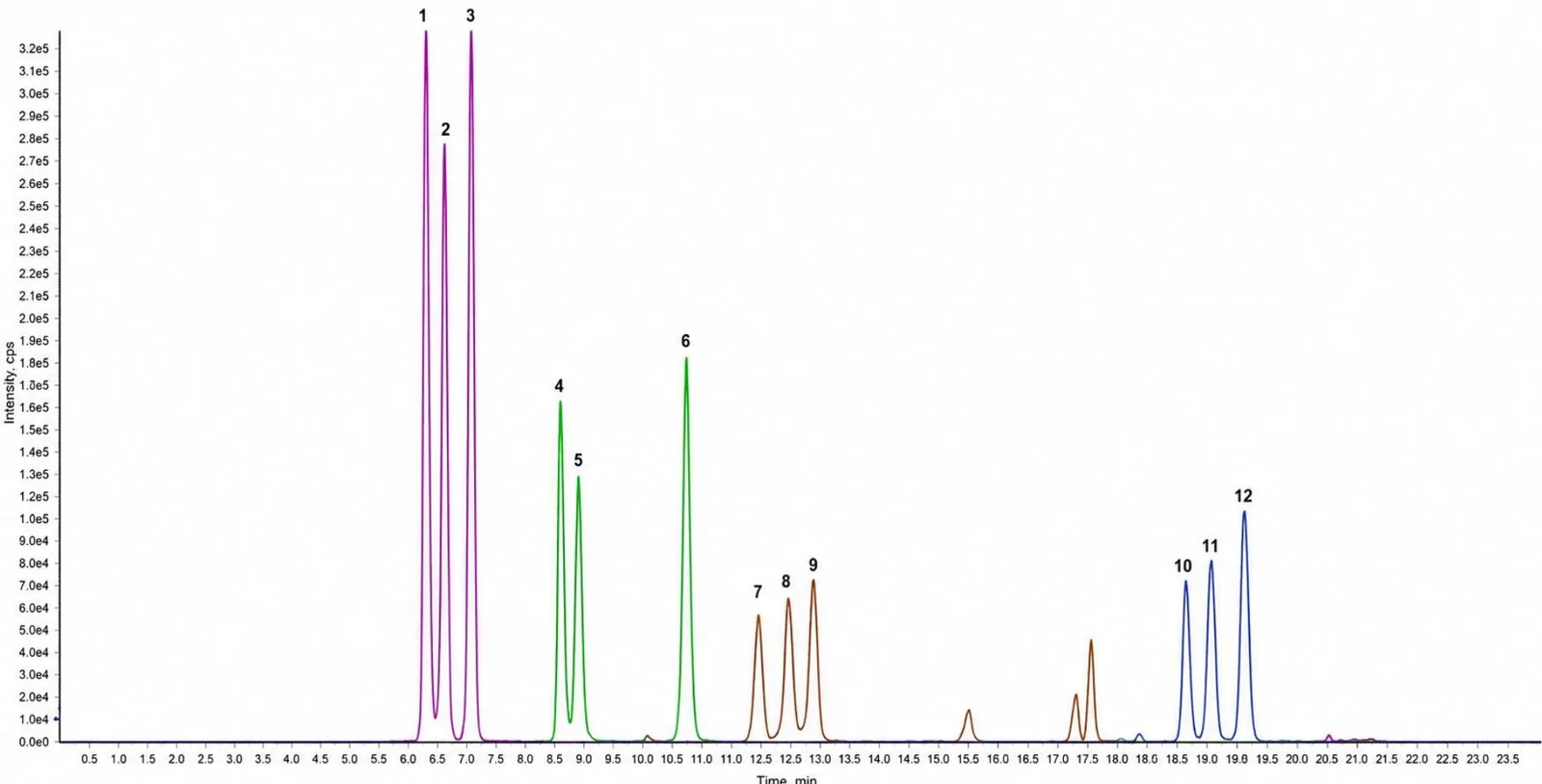
Extracted ion chromatogram of: 1 – intermedine N-oxide; 2 – lycopsamine N-oxide/indicine N-oxide; 3 – rinderine N-oxide/echinatine N-oxide; 4 – intermedine; 5 – lycopsamine/indicine; 6 – rinderine/echinatine; 7 – integerrimine N-oxide; 8 – senecionine N-oxide; 9 – senecivernine N-oxide; 10 – integerrimine; 11 – senecionine; and 12 – senecivernine

The sample preparation procedure was adapted from previously reported methods for PA analysis in honey (16, 17). However, because the earlier method included an N-oxide reduction step, the recoveries of N-oxides required optimisation in the present method. Increasing the ammonia volume in the SPE elution mixture proved to be a key factor, significantly improving N-oxide recoveries compared with the previous eluent composition while maintaining good recoveries of the basic PAs.

The expanded number of compounds covered in the current method and the modification of the SPE elution mixture recommended that the method was subjected to validation. Recoveries for all compounds were within the range of 80–120%. Repeatability and reproducibility values were ≤20%. The matrix effect, except for one compound, also fell within the acceptable range of 80–120%. The coefficients of determination exceeded the required threshold of 0.98, and back-calculation results were within ±20%, proving the linearity of the method. The lowest validation level of 1 μg/kg was taken as the LOQ for all PAs included in the method, as it provided established values for both accuracy and precision. Overall, all validation parameters fulfilled the SANTE document criteria (5).

The study results indicate the presence of PAs in various honeys. Of the 65 honey samples tested, 39 contained at least one of the analysed compounds, representing 60% of all samples. This suggests that PAs are common in honey, although in most cases, their concentrations were at very low levels dropping below the method’s LOQ (<1 μg/kg). As required by European Commission Regulations 2020/2040 and 2023/915, it is assumed that all values below the LOQ are zero (7, 8). Therefore, officially only 10 out of the 65 tested honeys should be considered positive samples. In these cases, concentrations expressed as the sum of detected PAs ranged from 1.0 to 46.9 μg/kg.

The honey with the highest content of PAs, amounting to 46.9 μg/kg, originated from the Mazowieckie voivodeship and was declared to be linden honey. In this case, alkaloids from the echimidine group were the main contributors to the contamination. These alkaloids are mainly produced by the plant *Echium vulgare* (commonly known as viper’s bugloss), and considering the profile of the detected compounds, *Echium vulgare* was the most probable source of the contamination (3). Another honey sample containing an elevated alkaloid concentration (23.6 μg/kg) originated from the Małopolskie region and was described as honey from forested areas. Seneciphylline was identified as the main PA in the contamination. In contrast, in the case of cultivated honeys, such as the tested rapeseed honey, PAs were mostly not detected above the method’s LOQ. Therefore, it can be inferred that the location of beehives may play an important role in the contamination of honey with pyrrolizidine alkaloids.

The remaining eight honey samples with quantified content contained relatively low alkaloid concentrations, ranging from 1.0 to 17.8 μg/kg, and originated in three cases from the Lubelskie, in two cases from the Mazowieckie, and in one case each from the Warmińsko-Mazurskie and Lubuskie voivodeships.

When assessing the individual compounds, the highest concentration was observed for seneciphylline (21.3 μg/kg). Quantifiable concentrations were also determined for heliosupine N-oxide (18.9 μg/kg), heliosupine (13.2 μg/kg), rinderine/echinatine (12.5 μg/kg) and echimidine (9.1 μg/kg). In contrast, the majority of detected compounds were present only at lower levels, typically ranging from 1.0 to 2.0 μg/kg.

The most frequently detected alkaloids were spartioidine and seneciphylline, which belong to the group of macrocyclic diesters. These alkaloids are known for their toxic properties, which may raise concerns among consumers regarding the safety of honey consumption if detected at elevated levels. However, the detected levels of macrocyclic diesters should not pose any risk to consumers as they were very low and mostly below the LOQ. The presence of other compounds, such as those from the intermedine group, might also be an important point of analysis, as they are open-chain monoesters, consumption of which may also have health consequences, even though it is well known that the open-chain monoesters are less toxic then the macrocyclic structures. It is worth noting that among the identified alkaloids, a significant portion occurred in the form of free bases rather than N-oxide forms, which is likely related to the breakdown of N-oxides due to the action of enzymes present in honey (13).

The findings of this study are consistent with previous reports on PA contamination in Polish honey. Earlier studies documented total PA concentrations ranging from 1.4 to 52.4 μg/kg, with mean and median values of 8.6 and 4.1 μg/kg, respectively (16). More recent data from 2022 reported concentrations of 2.2–31.6 μg/kg, with mean and median values of 11.0 and 9.0 μg/kg, respectively (17). In the present study, the honey samples showed PA concentrations ranging from 1.0 to 46.9 μg/kg, with mean and median values of 12.7 and 7.9 μg/kg. These results indicate that PA contamination in Polish honey has remained relatively stable over time, with low variability across different years of collection. The concentration levels of PAs in Polish honey were also comparable with other reported contamination levels in honey of European origin (14, 19).

Regarding the individual alkaloids in the overall contamination, previous studies most frequently detected echimidine, lycopsamine, intermedine and senecionine (16, 17). Similarly, in the present study, alkaloids from the intermedine, echimidine and senecionine groups were commonly quantified. Alkaloids from the seneciphylline group were also present in many samples, although their concentrations were below the LOQ. This may explain some of the differences observed compared to a 2018 study, as the analytical method used previously had higher LOD and LOQ, potentially missing compounds present at very low concentrations (16).

The contamination profile of the alkaloids is also similar to that reported by Roncada *et al*. (22). Similarly, another study on Italian honey presented detected profiles and concentrations of PAs comparable to the presented results (19). A similar alkaloids profile was also determined in other European honeys, where echimidine, lycopsamine, intermedine and senecionine-type PAs were the most often detected (14).

To assess consumer risk, the proposed 237 μg/kg body weight (bw) per day benchmark dose lower confidence level for a 10% response, defined as the lower bound of the 95% confidence interval for the dose associated with a 10% increase in cancer risk, was used as a reference point (8). To establish a daily intake limit of PAs that would not pose a health concern, a margin of exposure of 10,000 was applied. Based on this approach, the maximum allowable intake of PAs should not exceed 0.0237 μg/kg bw per day (2). Assuming an average honey consumption of 20 g per day (approximately one tablespoon), along with an average adult body weight of 60 kg and a child’s average body weight of 20 kg, the corresponding “safe” PA concentration in honey would be 71.1 μg/kg for adults and 24.2 μg/kg for children.

Considering the results obtained for PA content in honey from Poland, it can be seen that only one honey sample contained PA concentrations higher than those considered safe, but that consumption of such honey could pose a risk of adverse health effects, especially on children. The remaining honey samples should not carry a risk of adverse health effects and can be considered safe despite containing PAs.

## Conclusion

A method for the determination of 42 PAs was developed and validated in house. All validation parameters complied with the requirements of SANTE/11312/2021, demonstrating the robustness and utility of the method.

Sixty-five honey samples collected across Poland were analysed with the method. Detected PA concentrations were relatively low. The most common alkaloids were spartioidine and seneciphylline, followed by intermedine and echimidine. The analytical scope was broader than that of earlier studies, but neither the levels nor the qualitative composition of PAs in Polish honeys changed substantially. From a consumer risk perspective, the intake of PAs from nearly all the tested honeys would not cause adverse health effects. Only one sample indicated intoxicating potential, and all other honeys, could be considered safe for consumption although they contained measurable amounts of PAs.
